# Generation of diploid *Pichia pastoris* strains by mating and their application for recombinant protein production

**DOI:** 10.1186/1475-2859-11-91

**Published:** 2012-07-02

**Authors:** Ming-Tang Chen, Song Lin, Ishaan Shandil, Dewan Andrews, Terrance A Stadheim, Byung-Kwon Choi

**Affiliations:** 1 GlycoFi, Biologics Discovery, Merck Research Laboratories, Merck & Co., Inc, 21 Lafayette Street, Suite 200, Lebanon, NH, 03766, USA

**Keywords:** Mating, Diploid, *Pichia pastoris*, Strain stability, Fermentation, Recombinant protein expression

## Abstract

**Background:**

Yeast mating provides an efficient means for strain and library construction. However, biotechnological applications of mating in the methylotrophic yeast *Pichia pastoris* have been hampered because of concerns about strain stability of *P. pastoris* diploids. The aim of the study reported here is to investigate heterologous protein expression in diploid *P. pastoris* strains and to evaluate diploid strain stability using high cell density fermentation processes.

**Results:**

By using a monoclonal antibody as a target protein, we demonstrate that recombinant protein production in both wild-type and glycoengineered *P. pastoris* diploids is stable and efficient during a nutrient rich shake flask cultivation. When diploid strains were cultivated under bioreactor conditions, sporulation was observed. Nevertheless, both wild-type and glycoengineered *P. pastoris* diploids showed robust productivity and secreted recombinant antibody of high quality. Specifically, the yeast culture maintained a diploid state for 240 h post-induction phase while protein titer and N-linked glycosylation profiles were comparable to that of a haploid strain expressing the same antibody. As an application of mating, we also constructed an antibody display library and used mating to generate novel full-length antibody sequences*.*

**Conclusions:**

To the best of our knowledge, this study reports for the first time a comprehensive characterization of recombinant protein expression and fermentation using diploid *P. pastoris* strains. Data presented here support the use of mating for various applications including strain consolidation, variable-region glycosylation antibody display library, and process optimization.

## Background

The methylotrophic yeast *P. pastoris* has become an increasingly popular host for recombinant protein expression in recent times. As a eukaryote, *P. pastoris* has the capability to perform various post-translational modifications such as glycosylation, disulphide isomerization, proteolytic processing, and secretes correctly folded protein into culture media. *P. pastoris* can grow in methanol to very high cell densities in bioreactors, exceeding 450 g/L wet cell weight (WCW). Being an obligate aerobe when fed with methanol, *P. pastoris* does not switch to anaerobic metabolism that would lead to toxic metabolite accumulation under oxygen limited condition. This makes it possible to run high cell density fermentations under dissolved oxygen controlled processes.

Other benefits of the *P. pastoris* system include ease of genetic manipulation, stable expression, rapid cell growth, low-cost scalable fermentation processes and little to no risk of human pathogenic virus contamination. The *P. pastoris* system has been successfully used to produce a wide variety of heterologous proteins [1]. Fermentation titers at grams per liter scale have been reported for several target proteins including full-length antibodies [2-6].

In yeasts, the outer oligosaccharide chains of secreted proteins are decorated with high mannose type glycans. *P. pastoris*-derived glycosylated proteins are therefore potentially immunogenic. To overcome this issue, we pioneered a glycoengineered humanized *P. pastoris* expression system that could produce glycoproteins with glycosylation profiles similar to mammalian systems [7-13]. Therapeutic glycoproteins produced by the humanized *P. pastoris* platform have shown comparable folding, stability, and *in vitro* and *in vivo* efficacies in preclinical models to their counterparts produced from the CHO platform [14-16].

Like *S. cerevisiaeP. pastoris* is an ascomycetous homothallic budding yeast that can exist in both haploid and diploid states. Most industrial yeasts are diploids or polyploids. Diploid *S. cerevisiae* strains are generally considered to have greater thermo-stability as well as a higher tolerance to acid, ethanol, and other fermentation inhibitors than haploid strains [17,18]. Breeding polyploid industrial yeast strains has been shown to improve ethanol productivity and protein production [19]. Moreover, mating of *S. cerevisiae* has been successfully employed in other biotechnology and discovery applications such as yeast two-hybrid libraries [20] and antibody Fab display libraries [21]. In the case of an antibody Fab mating library, small variable heavy and light chain libraries are built and transformed separately into two haploid yeast strains with opposite mating types. Through mating of heavy and light chain haploid libraries, a large combinatorial Fab library can be generated and displayed on the diploid yeast surface [21].

One of the major differences distinguishing *P. pastoris* and *S. cerevisiae* mating is that *P. pastoris* is most stable in the vegetative haploid state and remains haploid unless forced to mate under certain conditions such as nitrogen limited-starvation [22]. The mated diploid yeasts efficiently undergo meiosis, sporulation, and switch back to the haploid state upon nitrogen limitation and other nutritional stresses. Due to the concern about diploid stability, especially in bioreactor fermentation processes, until now, no strategies have been described to utilize, much less to comprehensively quantify, recombinant protein expression and fermentation using diploid *P. pastoris* strains.

By using an IgG1 monoclonal antibody as the target protein, here, we demonstrate that both wild-type and glyco-engineered *P. pastoris* diploids provide stable and efficient heterologous protein expression in a nutrient rich shake flask environment. When the diploid strains were run in simple fed-batch, carbon-limited fermentation processes, both wild-type and glyco-engineered diploid strains afforded high protein productivity for at least 240 hours post-induction. Despite the observation of sporulation events happening during fermentation, we provide evidence showing that the majority of the yeast population maintained diploids in the 240 hour methanol induction. Finally, we compare recombinant protein productivities between a *P. pastoris* haploid IgG1 production strain and its isogenic, homozygous diploid clone. We show that diploid *P. pastoris* offers comparable protein productivity and N-glycosylation profile to its haploid counterpart.

As an application of mating *P. pastoris* haploids, we used mating to evaluate the ability of two libraries, one light chain and one heavy chain to produce unique functional antibody leads. The successful development of a *P. pastoris* display mating library can streamline the mAb discovery, maturation, and production in the humanized glycoengineered *P. pastoris* platform, enabling simultaneous screening of high affinity and satisfactory expression antibody leads. As a proof-of-concept study, we started with an IgG1 with a known affinity for its antigen and designed a small sequence variation library focusing on heavy-chain and light-chain CDR3 regions. The library contained >4,000 heavy-chain variants and >1,200 light-chain variants. Through mating of heavy and light chain *P. pastoris* haploid libraries, a diploid library containing more than 10^8^ colonies was successfully constructed, offering a greater than 10× coverage of the theoretical diversity.

## Results

### Construction of diploid *P. Pastoris* strains by mating

**Figure 1:**
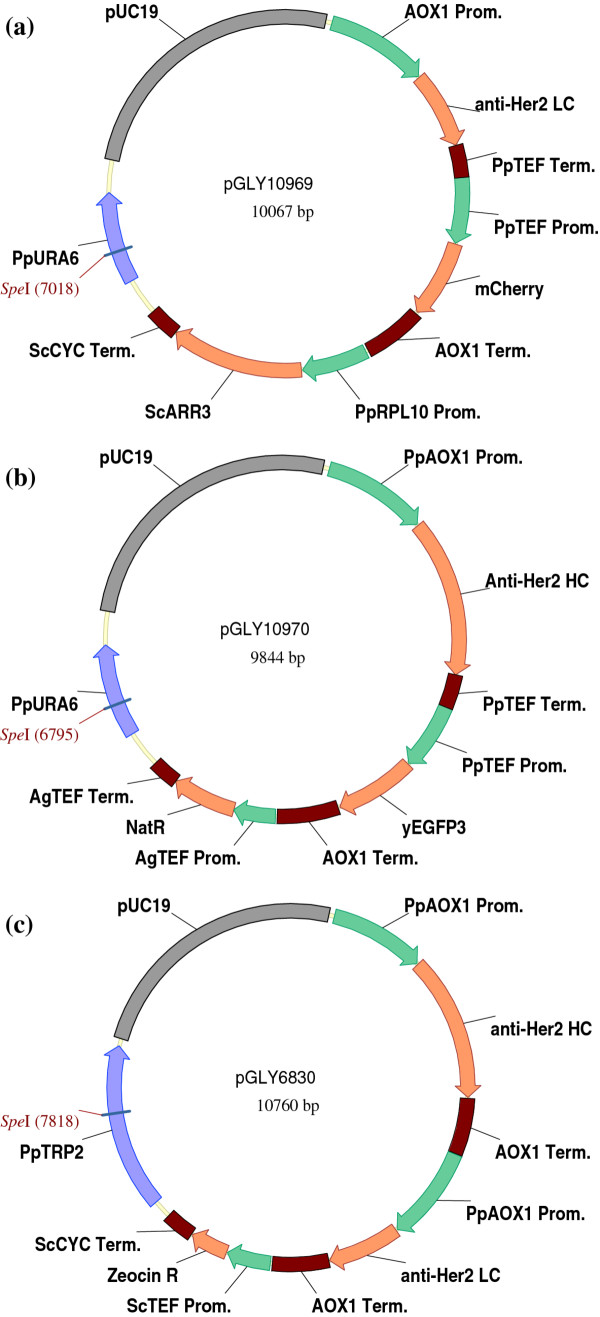
**Vectors for anti-HER2 light-chain and heavy chain expression.** (**a**) Plasmid pGLY10969 encodes the light chain of anti-HER2 antibody and mCherry red fluorescent protein in association with the arsenic resistance selection marker and targets the *URA6* locus in *P. pastoris* genome (**b**) Plasmid pGLY10970 encodes the heavy chain of anti-HER2 antibody and yEGFP3 green fluorescent protein in association with nourseothricin resistance selection marker and targets the *URA6* locus in *P. pastoris* genome. (**c**) Plasmid pGLY6830 encodes both light-chain and heavy-chain of anti-HER2 expression cassettes in association with zeocin resistance selection marker and targets to the *TRP2* locus in *P. pastoris* genome. The *Spe*I restriction enzyme site used to linearize each vector prior to yeast transformation is underlined.

To demonstrate the feasibility of using diploid *P. pastoris* strains for heterologous protein expression, we designed two expression vectors, pGLY10969 and pGLY10970 encoding the protein sequences for the light-chain and the heavy-chain of an anti-HER2 antibody, respectively. Their expressions are under the control of the AOX1 promoter and TEF transcriptional terminator (Figure 1a and 1b). To ensure the mating, two dominant selection markers are used, where plasmid pGLY10969 uses the *S. cerevisiae ARR3 gene* (ARS, confers resistance to arsenic) and plasmid pGLY10970 uses the *Streptomyces noursei* nourseothricin acetyltransferase gene (NAT, confers resistance to nourseothricin).

In order to understand the stability of diploids during methanol induction, plasmid pGLY10969 contains mCherry, a red fluorescent protein (RFP), and plasmid pGLY10970 includes yEGFP3, a green fluorescent protein (GFP), both regulated by the constitutive TEF promoter and the AOX1 transcription terminator. The plasmids also comprise the *URA6* ORF as an integration locus. By linearizing at the unique *Spe*I restriction site within *URA6* region, both vectors are integrated into the *URA6* genomic locus via single crossover.

**Figure 2:**
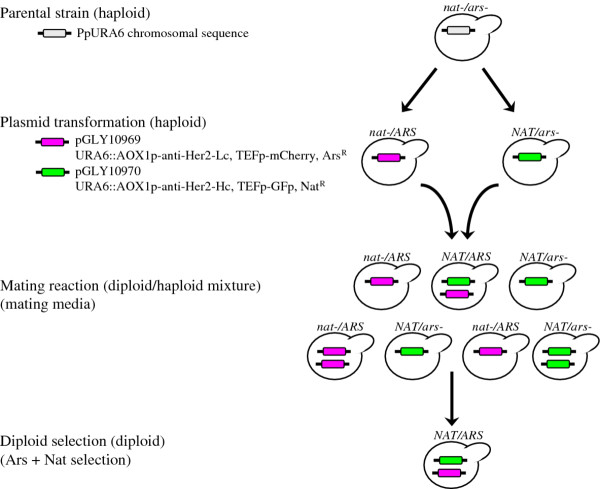
**Schematic overview of *P. pastoris *haploid strain construction, mating, and selection of anti-HER2 antibody expressing diploid strains.**

Table 1 lists the yeast strains used in this study. As shown in Figure 2, plasmids pGLY10969 and pGLY10970 were separately transformed into a parental strain to obtain two haploid strains. The diploid strains were constructed by mating these haploids followed by selection on plates containing nourseothricin and arsenic. Since both expression cassettes were integrated into the *URA6* locus, haploid progeny resulting from diploid sporulation could contain only one of the two expression cassettes. Consequently, only diploid cells were both nourseothricin and arsenic resistant (NAT^+^/ARS^+^), simultaneously expressed GFP and RFP, and secreted fully-assembled anti-HER2 antibody into the media. These features facilitated the monitoring of diploid population stability by using flow cytometry and selection plate methods, and were later used in the quantitative estimation of diploid population during the protein expression process.

**Table 1:** Yeast strains used in this study

**Strain**	**Ploidy**	**Parent**	**Plasmid**	**Glycan Structure**	**Genotype**
NRRL-Y11430	haploid	N/A	N/A	wild-type	OCH1 wild-type N-glycan *P. pastoris*
YGLY2-3	haploid	N/A	N/A	*och1Δ*	*och1-* N-glycan *P. pastoris*
YGLY8292	haploid	N/A	N/A	GS2.0 (Man5)	*och1-* glycoengineered GS2.0 starting strain*
YGLY8316	haploid	N/A	N/A	GS5.0 (G2)	*och1-* glycoengineered GS5.0 starting strain*
YGLY27429	haploid	NRRL-Y11430	pGLY10969	wild-type	URA6::AOX1p-anti-HER2-Lc, TEFp-mCherry, Ars
YGLY27430	haploid	NRRL-Y11430	pGLY10970	wild-type	URA6::AOX1p-anti-HER2-Hc, TEFp-GFP, Nat
YGLY27431	diploid	YGLY27429/YGLY27430	N/A	wild-type	URA6::AOX1p-anti-HER2-Lc, TEFp-mCherry, Ars
					URA6::AOX1p-anti-HER2-Hc, TEFp-GFP, Nat
YGLY27432	haploid	NRRL-Y11430	pGLY10969 pGLY10970	wild-type	URA6::AOX1p-anti-HER2-Lc, TEFp-mCherry, Ars
					URA6::AOX1p-anti-HER2-Hc, TEFp-GFP, Nat
YGLY27433	haploid	YGLY8292	pGLY10969	GS2.0 (Man5)	URA6::AOX1p-anti-HER2-Lc, TEFp-mCherry, Ars
YGLY27434	haploid	YGLY8292	pGLY10970	GS2.0 (Man5)	URA6::AOX1p-anti-HER2-Hc, TEFp-GFP, Nat
YGLY27435	diploid	YGLY27433/YGLY27434	N/A	GS2.0 (Man5)	URA6::AOX1p-anti-HER2-Lc, TEFp-mCherry, Ars
					URA6::AOX1p-anti-HER2-Hc, TEFp-GFP, Nat
YGLY27436	haploid	YGLY8292	pGLY10969 pGLY10970	GS2.0 (Man5)	URA6::AOX1p-anti-HER2-Lc, TEFp-mCherry, Ars
					URA6::AOX1p-anti-HER2-Hc, TEFp-GFP, Nat
YGLY13979	haploid	YGLY8316	pGLY6830	GS5.0 (G2)	TRP2::AOX1p-anti-HER2-Lc, AOX1p-anti-HER2-Hc, Zeocin
YGLY19313	haploid	YGLY13979	N/A	GS5.0 (G2)	TRP2::AOX1p-anti-HER2-Lc, AOX1p-anti-HER2-Hc, Zeocin, *ura5-*
YGLY19853	haploid	YGLY19313	pGLY24	GS5.0 (G2)	TRP2::AOX1p-anti-HER2-Lc, AOX1p-anti-HER2-Hc, Zeocin, *arg3-*
YGLY19895	diploid	YGLY19313/YGLY19853	N/A	GS5.0 (G2)	TRP2::AOX1p-anti-HER2-Lc, AOX1p-anti-HER2-Hc, Zeocin
					TRP2::AOX1p-anti-HER2-Lc, AOX1p-anti-HER2-Hc, Zeocin

*Detailed strain genotype is described in Materials and Methods.

Man5: Man_5_GlcNac_2_;

G2: Gal_2_GlcNAc_2_Man_3_GlcNAc_2_;

We used a filter-based mating protocol to determine the maximum mating efficiency in *P. pastoris*. We estimated that the maximum mating efficiency for the wild-type haploid *P. pastoris* strain (NRRL-Y11430) is about 10^-2^ (i.e. 1%, 1 diploid per 100 haploid cells) after 3 days, 25°C mating plate incubation. We reduplicated the filter mating experiment with *och1*Δ glyco-engineered strains and found that the mating efficiencies of both the *och1*Δ strain (YGLY2-3) and the GS2.0 strain (YGLY8292) are around 10^-3^ (i.e. 0.1%, 1 diploid per 1000 haploid cells). Not only did the *och1*Δ strains show a 10-fold decrease in maximum mating efficiency, but cells additionally required 5 days of mating plate incubation to reach maximum fusion (Table 2).

**Table 2:** **Mating efficiency of OCH1 wild-type and glycoengineered *P. pastoris *strains**

**Parental Haploid Strain**	**Glycan Structure**	**Mating Condition**	**Diploid Selection Markers**	**Estimated Maximum Mating Efficiency**
NRRL-Y11430	OCH1 wild-type	3 days, 25°C	ARS, NAT	10^-2^
NRRL-Y11430	OCH1 wild-type	3 days, 25°C	Arg1, His1	10^-2^
NRRL-Y11430	OCH1 wild-type	3 days, 25°C	Arg3, His1	10^-2^
NRRL-Y11430	OCH1 wild-type	3 days, 25°C	Arg3, His4	10^-2^
YGLY2-3	*och1*Δ	5 days, 25°C	ARS, NAT	10^-3^
YGLY2-3	*och1*Δ	5 days, 25°C	Zeocin, ARS	10^-3^
YGLY2-3	*och1*Δ	5 days, 25°C	Arg3, His1	10^-3^
YGLY8292	GS2.0 (Man5)	5 days, 25°C	Zeocin, ARS	10^-3^
YGLY8292	GS2.0 (Man5)	5 days, 25°C	Zeocin, NAT	10^-3^
YGLY8292	GS2.0 (Man5)	5 days, 25°C	ARS, NAT	10^-3^
YGLY8292	GS2.0 (Man5)	5 days, 25°C	Arg3, His1	10^-3^
YGLY8316	GS5.0 (G2)	5 days, 25°C	Zeocin, ARS	2.5 × 10^-4^
YGLY8316	GS5.0 (G2)	5 days, 25°C	Zeocin, NAT	2.5 × 10^-4^
YGLY8316	GS5.0 (G2)	5 days, 25°C	ARS, NAT	2.5 × 10^-4^
YGLY8316	GS5.0 (G2)	5 days, 25°C	Arg3, Ura5	10^-5^ *

*ura5- strain has a slow growth phenotype.

It should be noted that we calculated the mating efficiency based on the number of selective growth of crossed diploids using complementary markers. Because cells of the same strain could also mate, the total number of actual mating events can be up to twice the number reported here when self-mated diploids were included.

**Figure 3:**
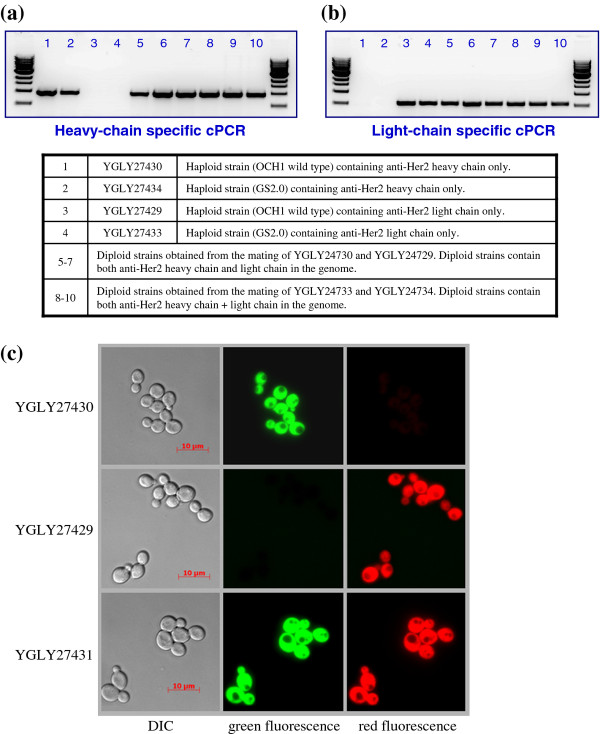
**PCR and microscopy confirmation of diploid polyploidy.** For **a**, and **b**, the primer sets listed in Table 3 were used to confirm the existence of anti-HER2 light and heavy chain expression cassettes in the diploid chromosome.

Confirmation of mated diploids was accomplished by colony PCR (cPCR) using the primer sets specific to the anti-HER2 light chain and heavy chain listed in Table 3. Figure 3 verifies that diploid strains contain genetic elements from both parental haploids. Lanes 1 and 2 of Figure 3a show that cPCR of the heavy-chain containing haploid strains (YGLY27430 and YGLY27434) produced a band using the heavy-chain specific primer set and no product was generated in light-chain containing haploid strains (YGLY27429 and YGLY27433). Likewise, lanes 3 and 4 of Figure 3b show that cPCR of the light-chain containing haploids (YGLY27429 and YGLY27433) produced a band using the light-chain specific primer set and no product was seen in heavy-chain only haploids.

**Table 3:** Primers used to confirm the existence of anti-HER2 light and heavy chains in diploid genome

**Reaction**	**Primer Name**	**Primer Sequence (5' to 3')**	**Primer Description**	**Product**
PCR-Hc	MT3	GGTTCCAATTGACAAGCTTTTGATTTTAACG	AOX1p_Forword	970 bp
	MT126	GTCCTCGTGAGAAACGTC	Anti_HER2_Hc_Reverse	
PCR-Lc	MT3	GGTTCCAATTGACAAGCTTTTGATTTTAACG	AOX1p_Forword	580 bp
	MT132	CTCTCTTGGGTAGAAGTTGTTCAACAA	Anti_HER2_Lc_Reverse	

Lanes 5–7 and 8–10 in Figure 3a and 3b indicate diploid colonies obtained from the mating of YGLY27430/YGLY27429 and YGLY27434/YGLY27433, where the presence of anti-HER2 heavy-chain and light-chain expression cassettes in the genome was confirmed by cPCR. Several hundred colonies from each mating reaction were analyzed by cPCR, revealing that more than 98% of colonies growing on selection media were diploid. Figure 3c displays representative microscopy fluorescence observations of wild-type haploid strains YGLY27430, YGLY27429 and their crossed diploid strain YGLY27431. Similar microscopy fluorescence was observed in the glycoengineered strain background (data not shown).

We repeated the mating experiments using different combinations of the dominant markers Zeocin, NAT, and ARS as well as the auxotrophic markers ARG1, ARG3, HIS1, HIS4, and URA5. In all tested mating circumstances, the mating efficiency in all *och1*Δ strains was found to be around 0.1% (Table 2), irrespective of the glycoengineered strains (GS2.0, and GS5.0). In wild-type *P. pastoris*, N-glycan structures consist mostly of Man_15-30_ with varying degrees of charged glycans. By comparison, N-glycans of *och1*Δ mutant strain are predominantly Man_8-12_, representing a noticeable shift to smaller glycans due to the lack of the outer chain [7]. Our results suggest that the knockout of *och1* decreases mating efficiency by 10 fold, likely because of decreased levels of mannose proteins in the *och1*Δ mutants, and that further glycoengineering has a negligible impact on mating fitness.

### Recombinant anti-HER2 production and diploid strain stability in methanol

**Figure 4:**
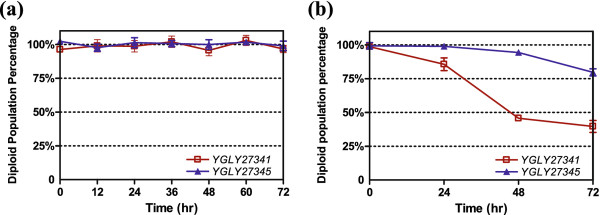
**Diploid strain stability analysis.** (**a**) Diploid strain stability showing total diploid population percentage in shake flasks followed by a methanol induction phase over 72 hour induction time course. (**b**) Time-dependent diploid stability in nitrogen-deprived sporulation media. For **a,** and **b**, percentage of diploid population was determined by the number of colonies growing on a diploid selection plate in comparison with the number of colonies growing in a non-selective plate. Symbols represent the average measurement values from triplicate experiments. As controls, parental haploid strains carrying either ARS or NAT selection marker did not form any colonies on selection plates containing both arsenic and nourseothricin (data not shown).

To understand heterologous protein production in diploid *P. pastoris* strains and diploid stability in methanol media, diploid yeasts YGLY27431 (wild-type) and YGLY27435 (*och1*, GS2.0) were induced in 2% BMMY methanol media for 72 hours in shake flasks for anti-HER2 production. Diploid strain stability was quantitatively determined by analyzing the percentage of diploid cells in the total cell population at 12 hr time intervals. The red curve in Figure 4a depicts the diploid population percentage of YGLY27431 over the 72 hour methanol induction period. The diploid population was maintained at around 100% throughout the entire methanol induction time. No obvious sporulation events were observed. Similarly, a stable diploid population was observed in the glycoengineered strain YGLY27435 (Figure 4a, blue curve).

We applied the same plate-based stability assay to examine diploid strain stability in nitrogen-deprived sporulation media. Figure 4b shows the diploid population in strains YGLY27431 and YGLY27435 in a nitrogen-deprived environment. A significant number of sporulation events were observed in the wild-type diploid strain YGLY27431 after 48 hrs in sporulation media (Figure 4b, red curve). After 72 h incubation, only about 40% of the cell population retained a diploid phenotype. Meanwhile, the glycoengineered diploid strain YGLY27435 (blue curve) appeared to sporulate at much lower frequency. This observation was consistent with a previous report in a *S. cerevisiae och1*Δ/*och1*Δ diploid strain where sporulation was reported to occur at a reduced efficiency [23].

It is worth mentioning that because *P. pastoris* asci contain up to 4 haploid ascospores, 1 diploid sporulation event might therefore generate up to 4 survival colonies on the non-selection plate if the spores are well separated. As a result, our plate-based stability curves reflect a relative but sensitive method to monitor diploid stability rather than absolute sporulation frequencies. In the case of Figure 4b data, 40% of the total diploid population could be caused by merely 25% of diploid cells undergoing sporulation, assuming that all *P. pastoris* spores were disjointed.

**Figure 5:**
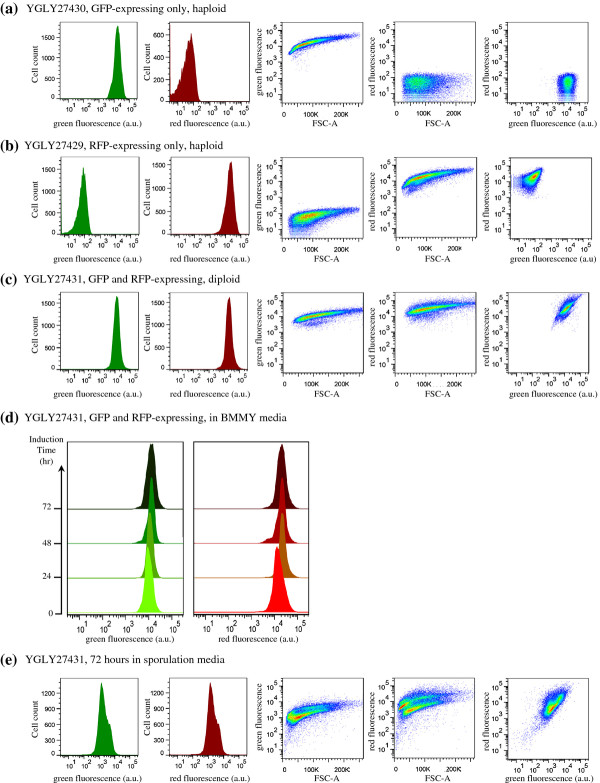
**Evaluation of diploid strain stability using flow cytometry analysis.** (**a**) Green and red fluorescence intensities of parental haploid yeast strain YGLY27430 (expressing only GFP). (**b**) Green and red fluorescence intensities of parental haploid yeast strain YGLY27429 (expressing only RFP). (**c**) Green and red fluorescence intensities of diploid yeast strain YGLY27431 (expressing both GFP and RFP). (**d**) Time course of green and red fluorescence intensities of diploid yeast strain YGLY27431 in shake-flask, methanol induction media. Fluorescence intensities were measured at 24-hour time intervals. (**e**) Fluorescence intensities of diploid yeast strain YGLY27431 after 72 hours cultivation in nitrogen-deprived sporulation media. For **a**, **b**, **c**, **d** and **e**, all yeast strains contained OCH1 wild-type N-glycan. Similar flow cytometry profiles were also observed in several different GS2.0 strain lineages YGLY27433, YGLY27434, and YGLY27435 (data not shown).

Diploid stability was also analyzed using flow cytometry. Figure 5a and 5b depict the green and red fluorescence profiles of the haploid yeast strains YGLY27430 (GFP-expressing only) and YGLY27429 (RFP-expressing only), respectively. Both haploids formed a distinct and highly fluorescent population distribution in their corresponding color channels. Figure 5c displays the fluorescence profiles of the diploid strain YGLY27431 and Figure 5d shows the fluorescence profiles of the diploid strain YGLY27431 under methanol induction over a time scale of 0–72 hours. Strain YGLY27431 retained strong GFP and RFP co-expression profiles throughout the 72 hour methanol induction period, suggesting that the diploid strains were stable under the conditions tested.

As a control experiment, when incubated for 72 hrs in nitrogen-deprived sporulation media, diploid cells formed two distinctive population distributions in both green and red fluorescence channels (Figure 5e). The high green fluorescence cell population corresponded to the vegetative diploid population, and the lower green fluorescence cell population could be explained by protein degradation during the sporulation process or lysis of the anucleate mother cell. Both green and red fluorescence profiles of the flow cytometry experiment provided reliable, real-time monitoring of diploid strain stability. Similar flow cytometry results were observed in the glycoengineered diploid strain YGLY27435 (data not shown).

### Bio-analytical characterization of anti-HER2 produced in diploid *P. Pastoris* strains

**Figure 6:**
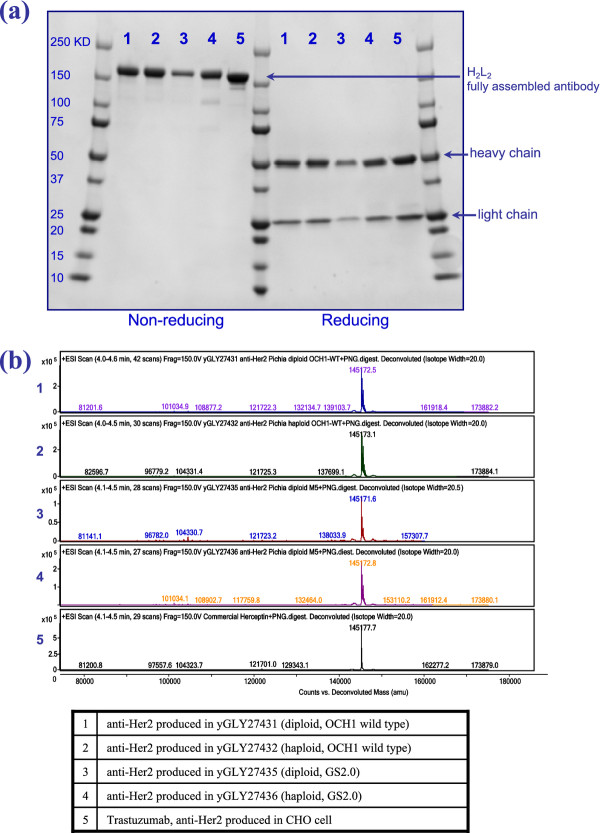
**Characterization of diploid *P. pastoris *-derived antibody.** (**a**) SDS-PAGE showing the quality of an anti-HER2 antibody produced in diploid *P. pastoris* strains in comparison with anti-HER2 material produced in haploid *P. pastoris* strains and their comparison with trastuzumab produced from CHO cells. (**b**) LC-MS mass spectrometry of anti-HER2 antibodies after release of N-glycan. For **a,** and **b,** anti-HER2 material produced in yeast strains was purified by affinity capture using protein A beads.

Figure 6a shows the reducing and non-reducing SDS-PAGE for anti-HER2 material generated by diploid *P. pastoris* strains and their comparison with anti-HER2 material generated by haploid *P. pastoris* strains and commercial trastuzumab. Anti-HER2 produced by diploid *P. pastoris* strains was well assembled and included the expected tetramer of two heavy and two light chains. An intact protein mass analysis was confirmed by liquid chromatography-mass spectrometry (LC-MS) after release of the N-glycan (Figure 6b). HER2 target binding of *P. pastoris* produced anti-HER2 was verified by surface plasmon resonance (Table 4). Similar kinetic binding constants were found for anti-HER2 produced in both diploid and haploid *P. pastoris* strains.

**Table 4:** **Kinetic binding constants of *P. pastoris *produced anti-HER2**

Samples	Description	*k*_a_ (1/M*s)	*k*_d_ (1/s)	*K*_D_ (nM)	Ave. *K*_D_ (nM)
YGLY27431	OCH1 wild-type, diploid	4.0E + 4	1.9E-4	4.7	4.63
YGLY27432	OCH1 wild-type, haploid	4.4E + 4	1.8E-4	4.2	
YGLY27435	GS2.0, diploid	4.1E + 4	2.2E-4	5.3	
YGLY27436	GS2.0, haploid	3.9E + 4	1.7E-4	4.3	

### Bioreactor cultivations of *P. Pastoris* diploids

**Figure 7:**
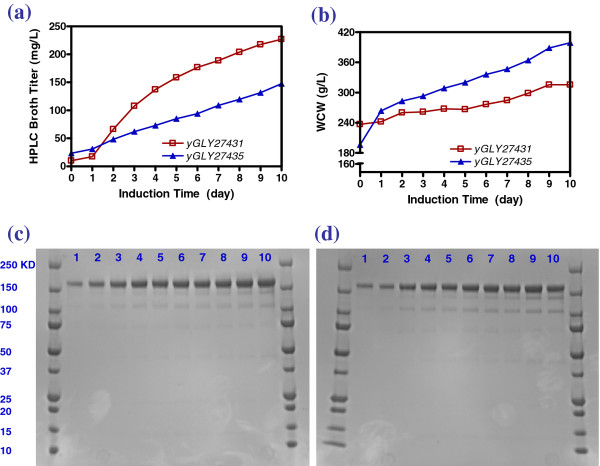
**Time course of protein productivity, biomass, and SDS-PAGE of diploid yeast strains YGLY27431 and YGLY27435 under fed-batch high-cell-density fermentation.** (**a**) Anti-HER2 productivity showing HPLC broth titer as a function of time. (**b**) Biomass showing WCW as a function of time. (**c**) Non-reducing SDS-PAGE of Protein-A captured YGLY27431 fermentation product. (**d**) Non-reducing SDS-PAGE of Protein-A captured YGLY27435 fermentation product.

We examined fermentability of diploid *P. pastoris* strains YGLY27431 (wild-type) and YGLY27435 (*och1*, GS2.0) using a simple fed-batch, carbon-limited 3 Liter fermentation platform. As shown in Figure 7a and 7b, both strains displayed an increase in antibody titer and WCW over a 10 day methanol induction time course. The anti-HER2 titers of diploid strains YGLY27431 and YGLY27435 at harvest were 227 mg/L and 148 mg/L, respectively under the suboptimal, constant feed rate fermentation conditions. The non-reducing SDS-PAGE in Figure 7c and 7d shows the high quality of fully assembled anti-HER2 material produced.

**Figure 8:**
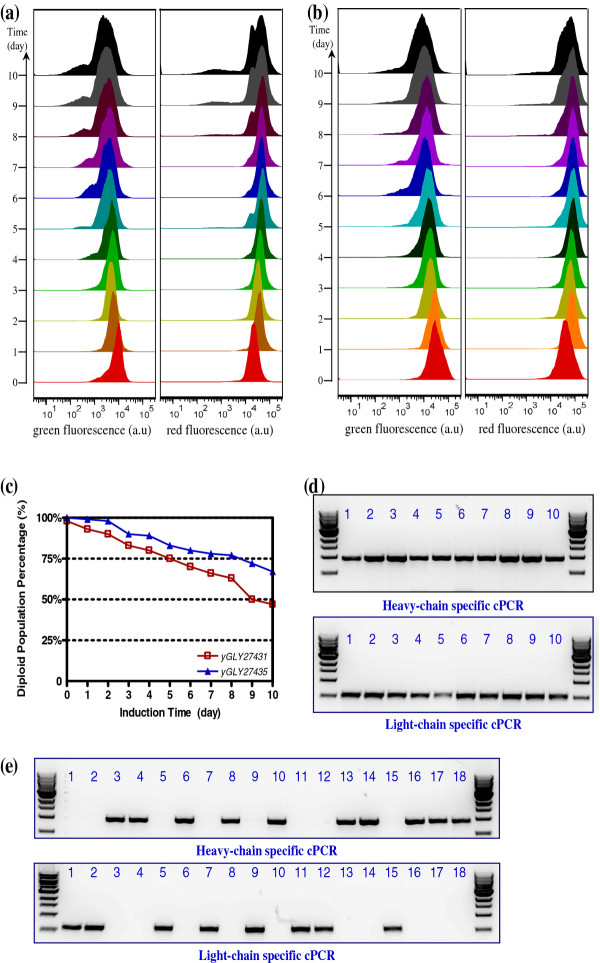
**Diploid strain stability in high-productivity fed-batch fermentation**. (**a**) Time course green and red fluorescence intensities of YGLY27431 showing OCH1 wild-type *P. pastoris* diploid strain stability over 10 days of induction. (**b**) Time course green and red fluorescence intensities of YGLY27435 showing glycoengineered -*P. pastoris* diploid strain stability over 10 days of induction. (**c**) Time-dependent diploid strain stability analyzed using a selection plates assay. (**d**) PCR confirmation of diploid genome. (**e**) PCR confirmation of sporulated haploid genome. For **a**, and **b**, fluorescence intensities were measured at 24-hour time interval using flow cytometry. For **c**, percentage of diploid population was determined by the number of colonies growing in diploid selection plate in comparison with the number of colonies growing in non-selective plates. For **d**, and **e**, the primer sets listed in Table 3 were used to confirm the existence of anti-HER2 light and heavy chain expression cassettes in yeast chromosome.

We analyzed diploid stability using both a plate-based stability assay and flow cytometry (Figure 8 a-c), and we observed a steady decrease in the diploid population (i.e. increasing sporulation) over time. After 10 days of fermentation, the percentage of diploids in the populations for YGLY27431 and YGLY27435 were 50% and 70%, respectively.

FACS analysis suggested that vegetative growing GFP or RFP expressing haploid populations in culture media were minimal. The sporulated diploids mostly existed in dormant spore states. Moreover, our previous experience showed that Protein A is able to capture heavy chain monomer. In Figures 7a and 7b, there is no obvious heavy chain monomer band after protein A purification, inferring that the haploids population is not significant.

We used cPCR to corroborate the existence of vegetative diploid and sporulated populations during fermentation. Figure 8d shows the cPCR results from 10 randomly picked colonies of YGLY27431 fermentation samples (10 days) growing on ARS/NAT selection plates. All colonies produced the PCR bands of anti-HER2 heavy and light chains, confirming their diploid genotype. Confirmation of ascospore formation in fermentation samples was done using diethyl ether extraction followed by cPCR. The cPCR result of 18 randomly picked colonies of diethyl ether treated YGLY27431 fermentation samples revealed that colonies contained either the heavy chain or light chain sequence in its genome but not both, confirming their haploidy. Similar cPCR results were observed in YGLY27435 fermentation samples (data not shown).

### Comparison of recombinant protein production between haploids and isogenic diploids

The above results show that both wild-type and glyco-engineered *P. pastoris* diploid strains are able to provide efficient and reliable anti-HER2 protein production in an established carbon-limited fermentation process. Our next aim is to perform a head-to-head assessment of recombinant protein production in a haploid *P. pastoris* production strain versus its isogenic homozygous diploid clone using N-glycan quality and protein titer as readouts. The haploid strain YGLY13979 was constructed by transforming plasmid pGLY6830 (Figure 1c) encoding both the light chain and heavy chain of anti-HER2 regulated by the AOX1 promoter into strain YGLY8316 [24]. Glycoproteins expressed in this strain were decorated with biantennary complex N-linked glycans.

We constructed YGLY19895, the isogenic homozygous diploid of YGLY13979, using URA5 and ARG3 auxotrophic markers as described in *Methods*. Table 5 lists the comparison of protein titer and glycan quality produced in strains YGLY13979 and YGLY19895 in a 1 L scale fermentor (n = 3). The diploid strain YGLY19895 had comparable broth titers to its haploid *P. pastoris* production counterpart (94% titer, within normal range deviation, n = 3). Moreover, anti-HER2 purified from the strains showed a very similar N-linked glycosylation profile (Table 5). Based on these observations, we suggest that diploid *P. pastoris* has the potential to produce proteins with high N-glycan quality.

**Table 5:** Fermentation profiles of haploid yeast strain YGLY13979 in comparison with its homozygous diploid strain YGLY19895

**Strain**	**Ploidy**	**Supernatant HPLC Titer (mg/L)**		**Broth Titer (mg/L)**		**WCW (g/L)**	**Induction Time (hr)**
YGLY13979	Haploid	1048		780		255	96
YGLY19895	Diploid			920		730		206	96
Strain	Ploidy	Man5%	G0%	G1%	G2%	Hybrid %	Complex %	
YGLY13979	Haploid	13.3	60.1	16.8	3.8	6.0	80.7	
YGLY19895	Diploid	15.6	59.1	15.1	2.5	7.7	76.7	

*Data represents the average measurement values from triplicate fermentation runs.

Man5: Man_5_GlcNac_2_;

G0: GlcNAc_2_Man_3_GlcNAc_2_;

G1: Gal_1_GlcNAc_2_Man_3_GlcNAc_2_

G2: Gal_2_GlcNAc_2_Man_3_GlcNAc_2_;

Hybrid: GlcNAc_1_Man_5_GlcNAc_2_ + Gal_1_GlcNAc_1_Man_5_GlcNAc_2_.

Complex: G0 + G1 + G2.

### Generation of a yeast antibody display library by mating

The construction of a yeast antibody display library by mating is dependent on the successful selection of appropriate strains and vectors. To facilitate large-scale diploid selection and cultivation of mated diploid cells, we chose the auxotrophic markers HIS1 and ARG1 for the selection of heavy and light chain vectors, respectively. As a proof-of-concept experiment, the host strain used was a *his1*Δ *arg1*Δ double auxotrophic wild-type strain. In this experiment, diploids were conveniently selected by growth in minimal liquid media without amino acid supplement. Compared with the strain selection using dominant drug markers, the use of auxotrophic makers eliminates the possibility of revertants that might arise through drug resistance.

We started with rationally designed heavy and light chain CDR3 variant libraries of an antibody with a known affinity for its antigen. The designed libraries contained >4,000 heavy-chain variants and >1,200 light-chain variants, where *S. cerevisiae* SED1 (GPI-cell wall glycoprotein) was used as a cell surface display anchor (Shaheen *et al.*, manuscript in preparation). Through mating of heavy and light chain *P. pastoris* haploid libraries, a diploid library containing more than 10^8^ colonies was successfully constructed and covered more than 10 times theoretical diversity.

We sequenced several hundred colonies, and all clones exhibited non-identical CDR3 sequences, supporting the diversity of the designed library (data not shown). In this proof-of-concept mating experiment conducted in wild-type *P. pastoris*, we estimated the mating efficiency to be around 0.25%. We successfully utilized this novel *P. pastoris* display system to identify a new antibody lead with improved biochemical characteristics and binding affinity using fluorescence activated cell sorting (manuscript in preparation). Our future plans include scaling up the current mating protocol to achieve a library with a diversity of 10^10^.

## Discussion

In a previous effort to humanize the protein glycosylation pathway in the yeast *P. pastoris*, we developed a systematic fusion library approach to select for the proper combination of targeting signal and catalytic domains. By sequentially screening several combinatorial genetic libraries to properly localize active eukaryotic mannosidases and glycosyltransferases, we were able to engineer the yeast glycosylation pathway to produce N-glycans with human-like glycan structures [7-13]. The genetic manipulations required for the construction of such strains were laborious and time-consuming.

Many species of yeasts, including *P. pastoris*, are mating competent. Mating enables two distinct haploid strains to fuse and generate a diploid strain possessing genetic elements from both parental haploids. Mating diploid yeast strains therefore potentially provides a convenient and faster route for strain construction and consolidation. Conventional experience that diploid *P. pastoris* strains are unstable suggests that after mating, immediate sporulation followed by screening stable haploid strains carrying desirable genetic constructs is imperative. Since the haploid *P. pastoris* genome contains only 4 chromosomes, gene integration loci are commonly linked to some degree and are presumably not well-segregated during sporulation. Strain construction using mating hence requires carefully chosen integration loci and the number of genes of interest can be limited by available selection markers.

By using protein titer and N-linked glycosylation profiles as quantitative readouts, we demonstrate that diploid *P. pastoris* strains offer comparable protein productivity and N-glycosylation profile to that of a haploid strain expressing the same antibody. Our comparison is based on isogenic homozygous diploid strains. Through careful selection of desired characteristics for mating, we consider it a possibility to construct heterozygous diploid *P. pastoris* strains possessing superior productivity and better stress tolerance than parental haploids. Polyploidy yeast strains may further be constructed through spheroplast fusion. This work lays a foundation for using mating for complex *P. pastoris* strain consolidation and direct screening of mated diploids for large-scale protein production. This method will be especially suitable for the systematic screening of combinatorial genetic libraries, and the number of genes of interest will not be limited by accessible selection markers.

Improved diploid bioreactor stability can be achieved through process and media optimization. In our experiments, both shake flask cultivation and fed batch fermentation were done using the same complex nutrient rich media. There were two major differences between shake flask and fermentation conditions: cell population density and media replenishment. In a shake flask, the start of induction cell population density was much lower than fed batch fermentation. This was indicated by a measured OD of 1–5 in shake flask versus 150–200 in fed batch fermentation. Additionally, culture media was replaced at start of induction in shake flask cultivation; while same media was carried over from growth phase into induction in fed batch fermentation. Due to both above stated reasons, the nutrient levels in fed batch fermentation would be lower than shake flask cultivation.

Based on available evidence, the authors believe that the addition of supplemental N-source can improve the performance of diploids in fed batch. Other fermentation modes such as perfusion and continuous culture may help in improving diploid stability. Alternatively, genetic engineering approach can be applied to obtain stable diploid *P. pastoris* cell lines. For example, the knockout of *IME1*, a key transcriptional regulator of early meiotic-specific genes, should abolish sporulation and stabilize diploid cell lines [25,26]. Considering that sporulation regulator genes are usually repressed under normal growth condition, we speculate that mutant *ime1*Δ, or other sporulation negative genes will have minimal effects on the AOX1 promoter activity or mating.

Yeast display of antibody mating libraries provides a practical and productive means for selecting higher affinity and well expressing antibodies using fluorescence activated cell sorting [27,28]. *S. cerevisiae* is presently the preferred expression host and has been successfully used for the isolation of antibody leads against a wide range of target antigens. However, the existence of fungal type N-glycans within the variable-region can still cause artifacts and have a negative impact on antibody selection [29]. Further development of the yeast antibody display system will require replacing fungal type N-linked glycans with mammalian-type N-glycans. With the uniform N-glycan profile generated by humanized *P. pastoris*, the glycoengineered *P. pastoris* display system provides a unique opportunity to leverage variable-region glycosylation in mAb discovery. An antibody display library containing N-linked glycosylation in variable-region can be rationally designed by introducing a consensus N-linked glycan motif (Asn-X-Ser or Thr, where X ≠ Pro) throughout the CDRs. N-link glycosylation can also be generated by using random mutagenesis approaches. We postulate that an antibody variable-region glycosylation library constructed like this will be especially suitable for affinity maturation, where antibodies with increased affinity for antigen are selected. We demonstrate that by utilizing *P. pastoris* mating it is feasible to generate relatively large yeast antibody libraries from easily constructed small libraries of heavy and light chains. Future development of glycoengineered *P. pastoris* system will ultimately enable a unique expression host, streamlining discovery, optimization, and production.

## Conclusions

The main purpose of this study is to investigate utilization of diploid *P. pastoris* strains for heterologous protein expression and to understand diploid strain stability in high-productivity fermentation. By using an anti-HER2 monoclonal antibody as a target protein, we demonstrate that recombinant protein production in both wild-type and glycoengineered *P. pastoris* diploids is stable and efficient using a nutrient rich shake flask cultivation. When the diploid strains were cultivated under bioreactor conditions, sporulation was observed. Still, both wild-type and glycoengineered *P. pastoris* diploids showed robust productivity and secreted recombinant antibody with high quality. We further demonstrate that diploid *P. pastoris* strains offer comparable protein productivity and N-glycosylation profile to that of a haploid strain expressing the same antibody.

During manuscript preparation, the authors found two U.S. patent applications describing the use of *Pichia pastoris* diploids submitted by another group of researchers [30,31]. Still, to the best of our knowledge, this study reports for the first time a comprehensive characterization of recombinant protein expression and fermentation using diploid *P. pastoris* strains. Data presented here support the use of mating for various applications including strain consolidation, variable-region glycosylation antibody display library, and process optimization.

## Methods

### Reagents & construction of anti-HER2 mAb expression plasmids

*Escherichia coli* strains TOP10 or XL10-Gold were used for recombinant DNA work. PNGase F, DNA restriction and DNA modification enzymes were obtained from New England BioLabs (Ipswich, MA). Figure 1 illustrates plasmid maps of anti-HER2 expression vectors used in this study. Anti-HER2 heavy and light chain genes (Herceptin® monograph, http://www.rochecanada.com) were codon optimized for *P. pastoris* expression and synthesized from GeneArt (Regensburg, Germany). Green fluorescent protein yEGFP3 [32] and red fluorescent protein mCherry [33] were codon optimized and synthesized from Genscript (Piscataway, NJ).

Plasmid pGLY10969 (Figure 1a) was a roll-in integration plasmid encoding the light chain of the anti-HER2 antibody and mCherry red fluorescent protein that targeted the *URA6* locus in *P. pastoris*. The antibody light chain expression cassette used methanol inducible AOX1 promoter and TEF transcription terminator sequences. *S. cerevisiae* alpha-mating factor signal sequences were applied to light chain as secretion signal peptide. The RFP expression cassette used constitutive TEF promoter and AOX1 transcription terminator sequences. The selection of transformants used arsenic resistance (ARS) encoded by the *S. cerevisiae ARR3* ORF under the control of the *P. pastoris* RPL10 promoter and *S. cerevisiae* CYC1 transcription terminator sequences. The plasmid further included a nucleic acid molecule for targeting the *URA6* locus.

Plasmid pGLY10970 (Figure 1b) was a roll-in integration plasmid encoding the heavy chain of anti-HER2 antibody and yEGFP3 green fluorescent protein that targeted the *URA6* locus in *P. pastoris*. The antibody heavy chain expression cassette used methanol inducible AOX1 promoter and TEF transcription terminator sequences. *S. cerevisiae* alpha-mating factor signal sequences were applied to heavy chain as secretion signal peptide. The GFP expression cassette used constitutive TEF promoter and AOX1 transcription terminator sequences. The selection of transformants used nourseothricin resistance encoded by the *Streptomyces noursei* nourseothricin acetyltransferase (NAT) ORF under the control of the *Ashbya gossypii* TEF1 promoter and *Ashbya gossypii* TEF1 terminator sequences. The plasmid further included a nucleic acid molecule for targeting the *URA6* locus.

Plasmid pGLY6830 (Figure 1c) was a roll-in integration plasmid encoding both the light and heavy chains of anti-HER2 antibody that target the *TRP2* locus in *P. pastoris*. Both the heavy and light chain expression cassettes used methanol inducible AOX1 promoter and AOX1 transcription terminator sequences. *S. cerevisiae* alpha-mating factor signal sequences were applied to both heavy and light chain as secretion signal peptide. For selecting transformants, the plasmid comprised an expression cassette encoding the Zeocin selection marker controlled by *S. cerevisiae* TEF promoter and *S. cerevisiae* CYC1 transcription terminator sequences. The plasmid further includes a nucleic acid molecule for targeting the *TRP2* locus.

### Yeast strains

Table 1 lists the yeast strains used in this study. All glycoengineered *P. pastoris* expression strains used were constructed from wild-type *P. pastoris* strain NRRL-Y11430 (Northern Regional Research Laboratories, Peoria, IL) using methods as described in [7,11,13].

The starting GS2.0 strain for the mating construction of recombinant anti-HER2 expression was YGLY8292 [*och1Δ, bmt1Δ, bmt2Δ, bmt3Δ, bmt4Δ, mnn4L1Δ, pno1Δ, mnn4Δ, Kluyveromyces lactis* &*Mus musculus* UDP-GlcNAc transporters, *Trichoderma reesei* α-1,2-MnsI]. Glycoproteins expressed from this strain lineage are decorated with Man5 glycans.

The starting GS5.0 glycoengineered strain for the construction of the recombinant anti-HER2 expression was YGLY8316 [24]. Glycoproteins expressed in this strain are decorated with biantennary complex, galactosylated (G2) glycans.

The plasmids encoding the light chain and heavy chain of the anti-HER2 antibody were linearized with *Spe*I to favor the integration at *TRP2* or *URA6* locus of *P. pastoris* genome and transformed into yeast by electroporation. Yeast strains YGLY27429 and YGLY27433 were generated by transforming pGLY10969, which encoded the light chain of anti-HER2 antibody and mCherry red fluorescent protein into *P. pastoris* wild-type strain NRRL-Y11430 and glycoengineered GS 2.0 strain YGLY8292, respectively. Yeast strains YGLY27430 and YGLY27434 were generated by transforming pGLY10970, which encoded the heavy chain of anti-HER2 antibody and GFP green fluorescent protein into wild-type strain NRRL-Y11430 and glycoengineered GS 2.0 strain YGLY8292, respectively.

Diploid yeast strain YGLY27431 was generated from the mating of YGLY27429 and YGLY27430; this strain contains *OCH1* wild-type N-glycan structure. GS2.0 diploid yeast strain YGLY27435 was generated from the mating of YGLY27433 and YGLY27434. Haploid anti-HER2 producing yeast strains YGLY27432 and YGLY27436 were obtained by double transformation of plasmids pGLY10969 and pGLY10979 into NRRL-Y11430 and YGLY8292, respectively. The strains comprised both anti-HER2 heavy and light chain expression cassettes and were used as anti-HER2 producing haploid control strains.

Yeast strain YGLY13979 [24] was generated by transforming pGLY6830, which encoded both heavy and light chains of anti-HER2 antibody, into the glycoengineered GS5.0 strain YGLY8316. The *URA5* gene of the prototroph YGLY13979 was flanked by two *lacZ* repeats introduced during the strain construction procedure*.*

Strain YGLY13979 was counter-selected in the presence of 5-fluoroorotic acid (5-FOA) to produce the yeast strain YGLY19313 in which the *URA5* gene was lost and only one copy of lacZ repeat remains in the chromosome. This rendered the strain auxotrophic for uracil. Afterwards, strain YGLY19313 was transformed with pGLY24 [34], a *ARG3* knockout vector using *URA5* as a selectable marker. This rendered the strain auxotrophic for arginine. Strain YGLY19853 was selected from the strains produced.

Diploid yeast strains YGLY19895 was generated from the mating of YGLY19313 and YGLY19853 using *ARG3* and *URA5* as selection markers. This strain, YGLY19895, is prototrophic and contains one copy of *URA5* and *ARG3* in its diploid genome. Strain YGLY19895 is considered to be an isogenic, homozygous diploid strain of YGLY13979.

### Mating and sporulation of *P. Pastoris*

Strains were mated using modified methods similar to as described in [35]. In general, twenty OD_600_ of cells from each parental haploid strain were combined and spread evenly onto a 100 mm mating Petri dish (0.5% sodium acetate, 1% potassium chloride, 1% glucose, 2% agar). For the *OCH1* wild-type *P. pastoris* strains, the mating reaction was allowed to proceed at 25°C for 3 days. For the glycoengineered strains, the mating reaction condition was 25°C for 5 days. Cells were then struck out to single colonies on diploid selection media. Glycoengineered diploid strains were selected on YSD plate containing 0.2 mM sodium arsenite and 25 μg/ml nourseothricin; *OCH1* wild-type *P. pastoris* diploids were selected with 0.5 mM sodium arsenite and 100 μg/ml nourseothricin.

The maximum mating efficiency for the *P. pastoris* strain tested was quantitatively determined using the filter method. In general, 1 OD_600_ of haploid *P. pastoris* cells corresponded to around 10^7^ cells. Five OD_600_ of cells from each parental haploid strain were combined and collected on the membrane surface (MF-Millipore^TM^ HAWP, mixed cellulose esters, hydrophilic, 0.45 μm pore, 47 mm diameter) using a vacuum filtration apparatus. The membrane was transferred cell side up to a 100 mm mating Petri dish. After 3 days, 25°C incubation (5 days for glycoengineered strains), cells were washed away from the membrane surface. The measured cell re-suspension OD is usually within a 1–2 fold range of initial starting OD (i.e. 10 OD_600,_ 10^8^ cells), indicating that cells were not actively dividing on the mating membrane.

The mating efficiency was determined from the number of diploid colonies growing on serially diluted diploid selection plates divided by (i) 10^8^ (starting cell numbers) and then (ii) the “*fold range*” of cell re-suspension OD. The “*fold range*” is a number usually between 1.5 and 2. .

For the yeast display mating library construction, a *his1*Δ and *arg1*Δ double auxotrophically marked, *OCH1* wild-type *P. pastoris* strain was used as the host strain. The heavy chain and light chain libraries were transformed separately into the above *P. pastoris* host. Mating of heavy chain and light chain haploid libraries was conducted on an agar surface (without filter) using a 222 mm × 222 mm Q-Trays Petri dish (Genetix – Molecular Devices, Inc).

One-hundred OD_600_ of cells from each parental haploid strain were combined and evenly spread on the mating media agar containing 50 μg/ml histidine and arginine supplement. After 3 days, 25°C incubation, cells were washed away from the plate surface and the diploid antibody display library was selected in liquid minimal media without amino acids. About 5 × 10^6^ diploid colonies were obtained after one mating reaction. The calculated mating efficiency here was around 0.25%.

To initiate diploid sporulation, about 50 OD_600_ of freshly patched diploids cells were resuspended in 10 mL liquid sporulation media (0.5% sodium acetate, 1% potassium chloride, 1% glucose) in a 50 mL conical tube (initial cell density = 5 OD_600_). The tube was incubated in a temperature-controlled shaker at 25°C and 200 rpm agitation. A random spore analysis procedure [35] was used to confirm ascospore formation in a fermentation sample. Vegetative diploid cells were eliminated by diethyl ether extraction and, afterwards, the resuspended spores were serially diluted and plated on YSD plates.

### Recombinant anti-HER2 antibody expression (shake-flask)

Yeast strains were analyzed for anti-HER2 production through small scale expression trials in 300 mL of buffered 2% glycerol complex medium (Teknova, Hollister, CA) in 1 liter shake flasks. Cell inoculums were incubated at 24°C for 2 days at 200 rpm (approximate OD_600_ = 1 to 5). Subsequently, the cells were pelleted by centrifugation (1474 g for 5 min) and resuspended in 300 ml of buffered 2% methanol-complex medium (BMMY) for induction of the AOX1 promoter. Pmt inhibitor PMTi4 in 100% methanol were added to the induction media to a final concentration of 1.6 μg/mL to minimize O-linked glycosylation [36]. The induction time was carried out at 24°C for 3 days with continual shaking at 200 rpm. The culture medium was cleared of yeast cells by centrifugation at 15000 g for 5 min.

### Bioreactor cultivations ─ carbon-limited fermentation

Bioreactor cultivations were performed in both 1 liter Fedbatch-pro, DASGIP BioTools or 3 liter Applikon glass bioreactors using the protocols described earlier [6]. In the present study, Martone was replaced with Soytone.

### Purification of anti-HER2 mAb

Anti-HER2 mAb was purified using a one-step, 96-well format STREAMLINE rProtein A method as described in [37].

### SDS–polyacrylamide gel electrophoresis (SDS–PAGE)

SDS–PAGE was carried out according to the Laemmli method with modified 4× non-reducing sample buffer (250 mM Tris, pH 6.8, 8% SDS, 40% v/v glycerol, 0.4% Bromophenol Blue sodium salt, 100 mM N-ethylmaleimide) and 4× reducing sample buffer (250 mM Tris, pH 6.8, 8% SDS, 40% v/v glycerol, 0.4% Bromophenol Blue sodium salt, 20% v/v b-mercaptoethanol). Gels were stained with 0.025% Coomassie Brilliant Blue R 250 in 7% v/v acetic acid, 40% v/v methanol and destained for 60 min in 10% v/v acetic acid.

### Binding kinetic analysis with her2 extracellular domain (ECD) protein

Surface plasmon resonance measurements of binding affinity using BIAcore T100 (Biosystems & ABI) were performed as described in [15].

### Liquid chromatography-mass spectrometry (LC-MS) analysis

LC-MS analysis was performed on an Agilent 6520 Q-TOF mass spectrometer connected to an Agilent 1200 HPLC system (Agilent Technologies, Santa Clara, CA). Prior to LC-MS analysis, samples were deglycosylated by treatment with PNGase F (New England Biolabs, Ipswich, MA) at 37°C for 2 hours. A Mass PREP Micro Desalting column (Waters, Bedford, MA) was used to remove the salts and trap the proteins in all the experiments.

The mobile phases were 0.1% formic acid in 100% HPLC water (Buffer A) and 0.1% formic acid in 90% acetonitrile/10% HPLC water (Buffer B) (Fisher Scientific). The LC gradient started with 100% Buffer A at a flow rate of 1 ml/min for 2 minutes to remove salts in the sample, and the flow was diverted to the waste line. Bound proteins were eluted by running 100% Buffer B at a flow rate of 0.4 ml/min for 5 minutes, flow was delivered to the mass spectrometer for analysis. The column was equilibrated by flowing through Buffer A at 1 ml/min for 3 minutes. Mass spectra were recorded during protein elution at a range of 600–3000 m/z. The mass spectrometer was operated in positive mode, the V_cap_ was set at 4500 V, and the gas flow rate was set at 13 L/min at 350oC. Data was analyzed using Agilent MassHunter Workstation Software (Agilent Technologies, Santa Clara, CA).

### Flow cytometry & microscopy

Flow cytometric analysis was performed using a BD LSRFortessa cell analyzer equipped with 488-nm and 561-nm excitation lasers. Manufacture recommended doublet discrimination gates in the dot plot with FSC and SSC were applied to ensure a population of single cells for analysis. The fluorescence intensities of the population were measured with a 530/30 nm optical filter for GFP and with a 610/20 nm optical filter for RFP in histograms. For each sample, 50,000 events were collected, and FlowJo software was used for the analysis of flow cytometry data.

Fluorescent microscopy observations were carried out using a Zeiss Axio Observer Z1 epifluorescence microscope equipped with a 1392 × 1040 pixel cooled CoolSNAP HQ2 CCD camera (Photometrics Corporation) and a 100× objective. False coloring was performed by capturing images with the appropriate colors (GFP filters: 436/20 excitation & 535/30 emission, RFP: 545/25 & 605/70).

### Glycan characterization

PNGase F, 2-aminobenzamide (2-AB) labeling and normal phase high performance liquid chromatography (HPLC) analysis of glycans were performed as described in [11].

### Protein A HPLC

Fermentation titer of anti-HER2 IgG1 produced from *P. pastoris* was quantitatively determined using protein A high performance liquid chromatography (HPLC). Briefly, POROS®A perfusion chromatography column was connected to an Agilent 1200 series HPLC with Diode Array Detector (Santa Clara, CA, USA). The pure mAb standard and sample were injected into a pre-equilibrated column at flow rate of 1 mL/min, followed by washing with PBS, pH 7.4. Anti-HER2 mAb was eluted with elution buffer (26 mM H_3_PO_4_ in PBS, pH 2.4) and monitored by UV absorbance at 280 nm.

A linear standard curve was generated by plotting the integrated peak areas against the injected amounts of standards. The amount of recombinant IgG in injected samples was interpolated from the standard curve. Titer was calculated by dividing the amount of mAb by the injected sample volume.

## Abbreviations

mAb;Monoclonal antibodies; ARS;Arsenic resistance; NAT;Nourseothricin resistance; RFP:Red fluorescent protein; GFP:Green fuorescent protein; cPCR;Colony PCR; WCW;Wet cell weight.

## Competing interests

The authors declare competing financial interests.

## Authors’ contributions

M.T.C., T.A.S., and B.K.C. conceived of the ideas implemented in this study. M.T.C. designed and carried out the molecular biology experiments. B.K.C. supervised the project. M.T.C., T.A.S., and B.K.C. performed data analysis and wrote the paper. M.T.C. and S.L. carried out the flow cytometry assay. I.S. carried out the 3 Liter bioreactor cultivations and participated in HPLC analysis. D.A carried out the protein A purification and participated in HPLC analysis. All authors read and approved the final manuscript.
